# Genetic Characteristics of CRF01_AE Among Newly Diagnosed HIV-1-Infected 16- to 25-Year Olds in 3 Geographic Regions of Guangxi, China

**DOI:** 10.1097/MD.0000000000000894

**Published:** 2015-05-29

**Authors:** Jing Zhang, Zhi-Yong Shen, Zheng Li, Shu-Jia Liang, Cui He, Fu-Xiong Liang, Yi Feng, Jian-Jun Li, Yu-Hua Ruan, Yue-Jiao Zhou, Yi-Ming Shao, Hui Xing, Ling-Jie Liao

**Affiliations:** From the State Key Laboratory for Infectious Disease Prevention and Control, National Center for AIDS/STD Control and Prevention, Chinese Center for Disease Control and Prevention, Collaborative Innovation Center for Diagnosis and Treatment of Infectious Diseases, Beijing (JZ, ZL, CH, YF, Y-HR, Y-MS, HX, L-JL); and Guangxi Center for Disease Prevention and Control, Nanning, Guangxi Province, China(Z-YS, S-JL, F-XL, J-JL, Y-JZ).

## Abstract

The aim of this study was to analyze the distribution of clusters and drug resistance of CRF01_AE among newly diagnosed, treatment-naïve HIV-infected teenagers and young adults in 3 major HIV-affected geographic regions of Guangxi Province, including the cities of Hezhou, Liuzhou, and Nanning.

Samples were sequentially collected from newly diagnosed HIV-infected 16- to 25-year olds in these 3 regions from 2009 to 2013. The viral genome was extracted, and the partial *pol* gene was amplified and sequenced. Phylogenetic analyses were used to determine HIV-1 subtypes and CRF01_AE clusters. Transmitted drug resistance (TDR) mutations were identified using the 2009 WHO list of TDR mutations.

A total of 216 sequences were obtained from CRF01_AE strains, which accounted for 83.1% of the 260 genotyped samples, of which 36 were from Hezhou, 147 from Liuzhou, and 33 in Nanning. Most (83.3%, 180/216) were from heterosexuals, followed by injection drug users (5.6%), homosexuals (4.2%), and unknown risk group (6.9%). Based on phylogenetic analyses by the maximum likelihood method, 5 distinct clusters (cluster 1–5) were identified with 213 (98.6%) sequences, whereas 3 (1.4%) sequences were ungrouped. In Hezhou, 88.9% (32/36) of CRF01_AE infections were caused by cluster 2, and 11.1% (4/36) were caused by cluster 1. In Liuzhou, 83.0% (122/147) of the CRF01_AE strains were found in cluster 1, 11.6% (17/147) from cluster 2, 1.4% (2/147) from cluster 3, 2.7% (4/147) from cluster 4, and 0.7% (1/147) from cluster 5. The distribution of CRF01_AE clusters was more even in Nanning than it was in the other 2 regions, with 18.2% (6/33) from cluster 1, 36.3% (12/33) from cluster 2, 9.1% (3/33) from cluster 3, 18.2% (6/33) from cluster 4, and 12.1% (4/33) from cluster 5. The most frequent TDR mutations were M46I (2) in the protease region and Y181C (2) from the reverse transcriptase fragment.

Clusters 1 and 2 of CRF01_AE strains were prevalent in Liuzhou and Hezhou, respectively. However, multiple CRF01_AE clusters existed in Nanning. This can be partially explained by the high mobility of laborers in Nanning, the capital city of Guangxi. The prevalence of TDR was low.

## Abstract 2

Supplemental Digital Content is available in the text

## INTRODUCTION

CRF01_AE is one of the prevalent HIV-1 subtypes in the world,^1^ which originated from Central Africa, and spread epidemically in Asia.^2^ In Thailand and Vietnam, CRF01_AE accounts for more than 95% of HIV-1 infections.^3^ In China, the CRF01_AE strain was first identified among commercial sex workers who returned to Yunnan Province from Thailand in 1994,^4^ and it was found in intravenous drug users (IDUs) from Guangxi Province in 1996.^5^ During the following 10 years, it became the dominant HIV-1 strain in many provinces^6–8^ and was responsible for 27.5% in all HIV-1 infections.^9^ The rapid spread of CRF01_AE in many regions of China such as Beijing^10^ and Guangxi^11^ has drawn much attention.^12^

Guangxi, a southwest province, bordering Vietnam in the south, is considered to be one of the provinces with the most severe HIV-1 epidemic in China. The number of newly reported persons living with HIV-1 in Guangxi increased from 6989 in 2006 to 12,065 in 2009. The cumulative number of HIV-1-infected people in Liuzhou, Nanning, and Hezhou, ranked first, second, and fourth in Guangxi in 2009, respectively.^13^ HIV-1 in Guangxi has spread from IDUs^14^ into sexual transmissions^15^ in the last few years. In a recent survey, CRF01_AE was found to be the dominant strain, which was responsible for 72.7% of infections in Guangxi.^16^ In addition, CRF01_AE accounted for 80.1% of heterosexually transmitted HIV-1 cases.^15^ CRF01_AE has replaced CRF08_BC^17^ and has become the leading HIV-1 strain in Guangxi.^6,13,15,16,18^

According to recent reports in China, CRF01_AE has also led to a new epidemic in many provinces and municipalities, and displays a complex lineage map in many regions.^7^ In a survey conducted in Liaoning Province, CRF01_AE strains among men who have sex with men (MSMs) were grouped into 2 distinct clusters.^19^ However, CRF01_AE strains in Guizhou were distributed in 4 major clusters.^20^ Recent studies also found that CRF01_AE in Hong Kong likely originated from 3 separate clusters.^6^ Later, there were estimated to be at least 4 clusters in China,^12^ whereas the analyses of near-full-length genome (NFLG) sequences revealed 7 different CRF01_AE clusters in China.^21^ As a hot spot of the HIV-1 epidemic, Guangxi Province has CRF01_AE strains derived from multiple origins.^15,18^ However, most researches on CRF01_AE in Guangxi were limited to only 1 risk group, which might have missed other information. To gain a deeper understanding of the genetic characteristics of CRF01_AE, we conducted a study among newly diagnosed HIV-1-infected patients aged 16 to 25 years who were likely to have been recently infected.

## MATERIALS AND METHODS

### Study Population

All the study participants were from the surveys of transmitted drug resistance (TDR), which were carried out in cities of Hezhou, Liuzhou, Nanning, Guangxi Province from 2009 to 2013, according to an adapted WHO protocol. Briefly, subjects were newly diagnosed HIV-infected 16- to 25-year olds, and sequentially entered the surveys, at HIV testing centers of local Centers for Disease Control and Prevention (CDCs). Exclusion criteria were being HIV antibody positive in previous tests, having received antiretroviral therapy, CD4 <200 cells/μL, if CD4 counts were available. Individual counseling was provided and the demographic information was collected from all the HIV-positive participants through interview by trained local healthcare workers. This study was approved by the institutional review board (IRB) at the National Center for AIDS/STD Control and Prevention (NCAIDS), China CDC, and Guangxi CDC.

### RNA Extraction, Nested Reverse Transcriptase Polymerase Chain Reaction, and Sequencing

Remnant specimens of HIV testing or first CD4 counting were used for genotyping. Total RNA was extracted from 200-μL plasma samples by using the QIAamp Viral RNA Mini Kit (Qiagen, Valencia, CA) according to the manufacturer's protocol. The RNA template was then reverse transcribed to cDNA using primer RT21 (5′-CTGTATTTCTGCTATTAAGTCTTTTGATGGG-3′, 3509–3539). Nested PCR was used to amplify a 1.2-kb fragment of the *pol* gene from the cDNA template. This partial *pol* gene comprised the entire protease (PR) encoding region and the first 300 codons of the reverse transcriptase (RT) gene. The first-round PCR primers were MAW26 (5′-TTGGAAATGTGGAAAGGAAGGAC-3′, 2028–2050) and RT21. The second-round primers were PRO1 (5′-CAGAGCCAACAGCCCCACCA-3′, 2147–2166) and RT20 (5′-CTGCCAGTTCTAGCTCTGCTTC-3′, 3441–3462). The PCR products were purified by using a QIAquick Gel Extraction Kit, and sequenced with BigDye using ABI3730 (Life technology, Foster City, CA).

### Phylogenetic Analyses

PhyML 3.0 was used to construct a maximum likelihood phylogenetic tree with all of the sequences obtained, and tree topo logies were determined using subtree pruning and regrafting methods.^22^ The branch significance was analyzed by bootstrap with 500 replicates and inter-subject distances were calculated. The final tree was viewed using MEGA5.0 software and FigTree v1.3.1, as previously described.^23^ To determine the clusters, sequences of CRF01_AE strains from China and neighboring countries such as Thailand and Vietnam^21^ from the HIV database (http://hivweb.lanl.gov) were selected to be references. The serial number of clusters was defined according to the recent NFLG analyses.^21^

### Identification of TDR mutations

The obtained sequences were compared to the consensus B sequence, and TDR mutations were identified according to the 2009 WHO list of TDR mutations.

### Statistical Analysis

The categorical variables were calculated as absolute values and percentages, and were compared using *χ*^2^ or Fisher exact test. Statistical analyses were performed using SAS version 9.3 (SAS Institute, Cary, NC). All probability values were 2-tailed, and the statistical significance level was defined as *P* < 0.05.

## RESULTS

### Study Subjects

**FIGURE 1:**
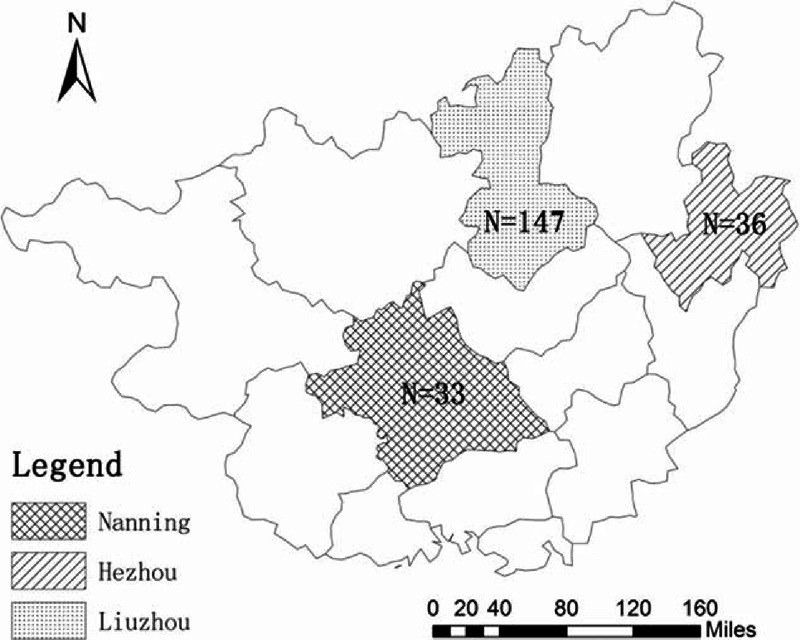
The geographical distribution of 216 HIV-1 CRF01_AE-infected people in 3 regions of Guangxi Province. The number of cases in each region is indicated as “N” in the map.

A total of 260 participants who were newly diagnosed, treatment-naïve, and 16 to 25 years of age in Guangxi Province entered the study. Among which, 37 were sequentially included in Hezhou from March 2009 to March 2010, 169 were consecutively included in Liuzhou from January 2011 to October 2013, and 54 were recruited in the order of HIV testing in Nanning from November 2012 to August 2013. According to phylogenetic analyses based on the partial *pol* fragment, CRF01_AE accounted for 83.1% (216/260) of the infections. Among the 216 samples with CRF01_AE strains, 36 were from Hezhou, 147 from Liuzhou, and 33 from Nanning (Figure 1). The mean age of these subjects was 23 years; 40.3% (87/216) were men (Table 1). A total of 115 (53.2%) participants were from the Han ethnic group, 71 (32.9%) were from the Zhuang ethnic group, and the remaining 30 participants were from other ethnic groups. The study subjects included 83.3% (180/216) heterosexuals, 5.6% (12/216) IDUs, 4.2% (9/216) homosexuals, and 6.9% (15/216) unknown. The risk populations of the study subjects were differently distributed in the 3 regions (*P* < 0.001). The highest proportion of heterosexuals was found in Liuzhou (89.8%, 132/147), and the largest number of homosexuals was found in Nanning (21.2%, 7/33).

**TABLE 1:**
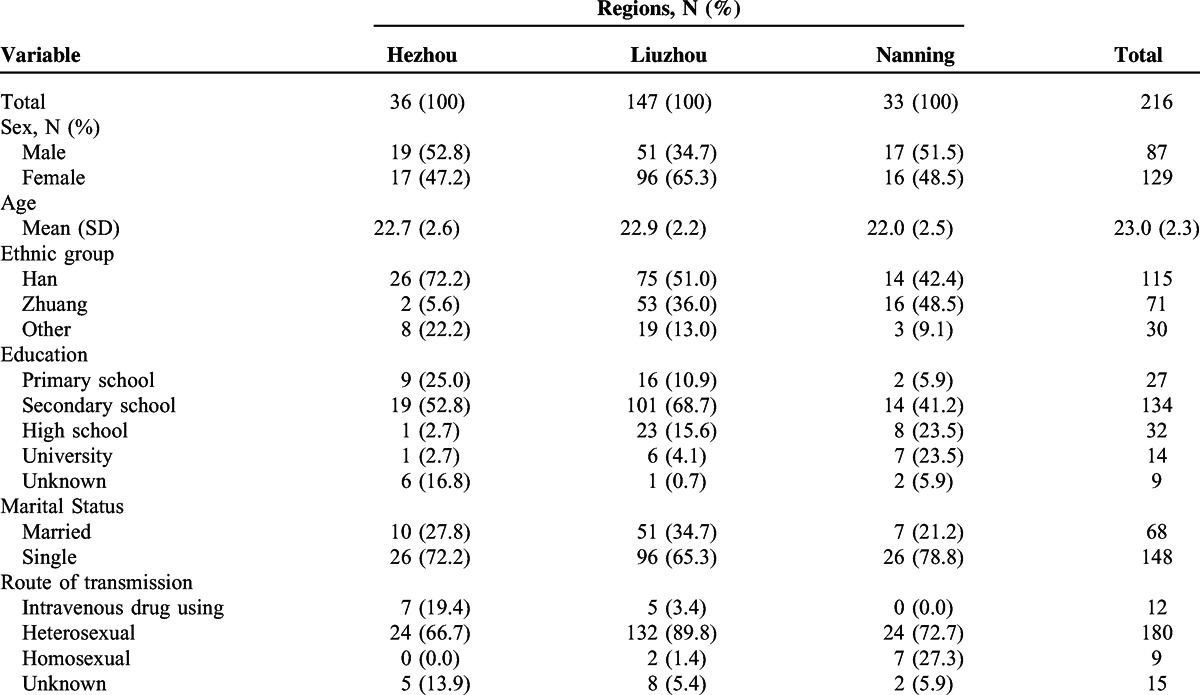
Characteristics of the Study Subjects Infected With the CRF01_AE Virus in 3 Regions of Guangxi Province

### HIV-1 CRF01_AE Clusters

**FIGURE 2:**
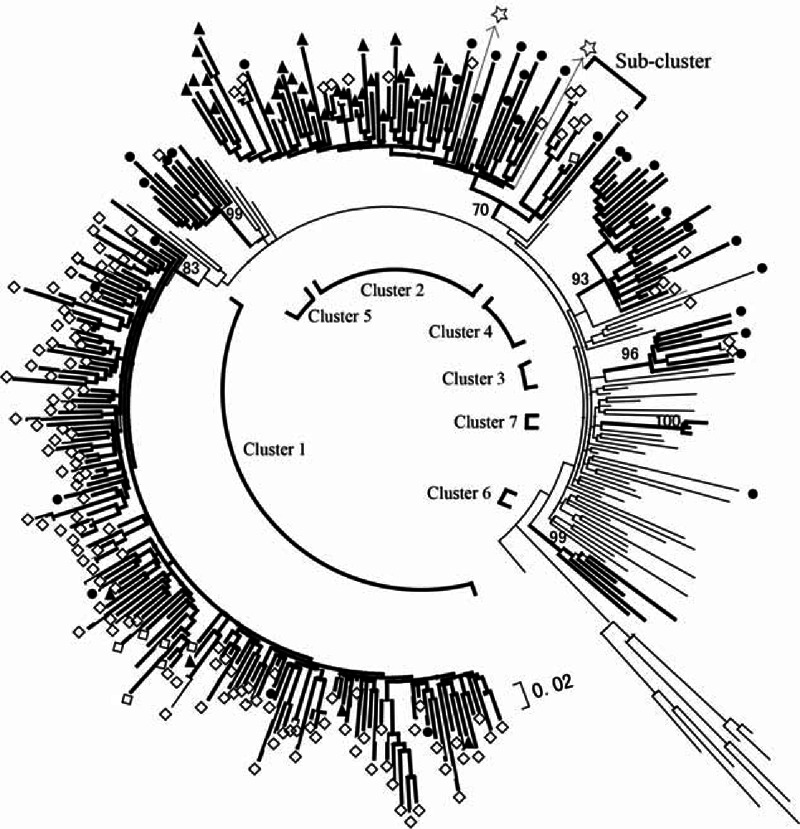
The phylogenetic tree constructed by PhyML 3.0 with the maximum likelihood method, based on the partial *pol* fragment. Thick and thin black branches represent those of study and reference sequences, respectively. Solid triangles (▴), hollow diamonds (⋄), and solid circles (●) represent the sequences from Hezhou, Liuzhou, and Nanning, respectively. Hollow 5-pointed stars (☆) are references from Vietnam. A sub-cluster was found in cluster 2. The branch significance was analyzed by bootstrap with 500 replicates and inter-subject distances were calculated. Only bootstrap values ≥70 are shown at the corresponding nodes. The scale bar represents 2% genetic distance (0.02 substitutions per site).

Based on phylogenetic analyses, 5 distinct clusters were identified with 213 (98.6%) sequences, whereas 3 (1.4%) sequences were ungrouped, as shown in Figure 2. The distribution of CRF01_AE cluster was significantly different among the 3 regions (*P* < 0.001). In Hezhou, 88.9% (32/36) of CRF01_AE sequences were in cluster 2, and 11.1% (4/36) of infections were in cluster 1. In Liuzhou, 83.0 % (122/147) of the CRF01_AE strains were in cluster 1, 11.6% (17/147) in cluster 2, 1.4% (2/147) in cluster 3, 2.7% (4/147) in cluster 4, and 0.7% (1/147) in cluster 5. The distribution of CRF01_AE clusters was more even in Nanning than in the other 2 regions, with 18.2% (6/33) in cluster 1, 36.3% (12/33) in cluster 2, 9.1% (3/33) in cluster 3, 18.2% (6/33) in cluster 4, and 12.1% (4/33) in cluster 5. Among the 5 clusters, cluster 1 had 132 members, whereas cluster 2 had 61 members. Cluster 1 strains were found among 118 heterosexuals, 2 homosexuals, 5 IDUs, and 7 unknown risk groups. Cluster 2 strains were found among 45 heterosexuals, 3 homosexuals, 7 IDUs, and 6 unknownrisk groups (Table s1, http://links.lww.com/MD/A283).

### Prevalence of TDR

The TDR mutations were found in 10 samples, with a prevalence of 4.6% (10/216), with 1.4%, 1.4%, and 2.3% of resistance to PIs, NRTIs, and NNRTIs, respectively. Two TDR mutations, M46I (2) and I50V (1) were found in the protease region, and the other eight TDR mutations, K65E(1), D67N(1), T69N(1), K103N(1), Y181C(2), G190E(1), L210W(1), and P225H(1) were from the reverse transcriptase fragment. All 5 drug-resistant strains from Liuzhou were in cluster 1. Four other drug-resistant strains, 2 strains from Hezhou and 2 from Nanning, belonged to cluster 2. The remaining 1 drug-resistant strain from Nanning was outside of the identified clusters.

## DISCUSSION

HIV-1 CRF01_AE, which predominates in sexual risk populations, is the prevalent CRF in the world, especially in Asia.^1,20^ Multiple lineages of CRF01_AE strains were introduced into China during early-middle 1990s.^21^ Recent studies have reported that CRF01_AE became the dominant subtype in Guangxi Province,^15,16,18^ which is consistent with the findings of our study. However, the proportions of CRF01_AE (83.1%) in this study are a little higher than previously published data, which found that the percentages of CRF01_AE were 72.7% in 254 cases from December 2012 to January 2013,^16^ and 80.1% in heterosexually transmitted HIV-1patients.^15^ In the present study, the HIV-1 CRF01_AE strains prevalent in heterosexually transmitted patients in Liuzhou and Hezhou were characterized.

Phylogenetic analysis of viral sequences has recently been used to define populations with HIV-1 infection.^11,24^ The phylogenetic tree in this study suggested that these CRF01_AE strains are present in high diversities in different risk groups and regions. In this study, 89.4% (118/132) of samples in cluster 1 were from heterosexuals. The proportion of heterosexuals was 73.8% (45/61) in cluster 2 and100.0% (5/5) in cluster 3. These findings are consistent with Feng et al's^21^ results that clusters 1, 2, and 3 were mainly found among heterosexuals.

Clusters 1 and 2 of CRF01_AE strains were prevalent in Liuzhou and Hezhou, respectively. Cluster 1 corresponded to cluster II in a previous study^18^ conducted in Guangxi Province. It was a new cluster of CRF01_AE that has spread rapidly in Guangxi Province.^15^ In this study, 92.4% (122/132) of cluster 1 sequences were from the Liuzhou strains and well grouped with some other Guangxi reference strains. This suggested that strains of cluster 1 in Liuzhou mainly originated from Guangxi and were internally transmitted. However, cluster 2, which corresponded to cluster I in Zeng et al's study,^18^ had been proved to have a close relationship with CRF01_AE strains from both Guangxi Province and Vietnam. Similar findings were found in other studies,^7,14^ which showed the existence of the Vietnam-China-related CRF01_AE lineage in Guangxi Province. It is noted that a sub-cluster was found within cluster 2, including 7 sequences from Liuzhou and 1 sequence from Nanning, which was not reliable, as the bootstrap value was 60, not high enough. However, it may suggest that a proportion of cluster 2 strains had been introduced into Liuzhou over a long period and might be in the process of forming a mono-lineage. In this study, multiple CRF01_AE clusters were detected in Nanning. Nanning is the capital city of Guangxi Province, which has a large population flow and complex population composition. The high mobility of laborers in Nanning can partially explain the multiple CRF01_AE clusters existing there.

All the 3 regions in this study showed transmitted HIV-1 drug resistance. The prevalence of drug resistance was 4.6% (10/218), higher than previously reported 3.2%.^16^ The drug-resistant strains were diffused in clusters, and did not bunch into a mono-lineage cluster. The most common TDR mutations were M46I in PR and Y181C in RT.

There are several limitations in this study. First, all of the samples were from individuals between 16 and 25 years and thus there was a lack of information about CRF01_AE in other age groups. Second, the geographic distribution in Guangxi Province was limited and the sample distribution in each region was unbalanced. Despite the large number of newly diagnosed HIV-infected peoples, those of 16 to 25 years of age were few in Guangxi. The surveys of TDR had to switch among regions with a higher HIV/AIDS epidemic. Thus, the results of this study may only reflect CRF01_AE strains prevailing at a few hot spots of HIV epidemic in Guangxi Province. Third, subjects in this study were not collected in the same period. Infected individuals of Hezhou were recruited in 2009 and 2010, samples of Liuzhou were collected from 2011 to 2013, whereas the strains from Nanning were collected in 2012 and 2013. Further study with more intense sampling in the same period may reveal more relationships of HIV-1 transmission between regions.

In conclusion, the different distributions of the CRF01_AE cluster were found in 3 main HIV-1-affected geographic regions of Guangxi Province, which meant that there were multiple origins of HIV-1 CRF01_AE transmission. Further study should be performed to reconstitute the epidemic pathway, and provide more suggestions for HIV/AIDS control and prevention in Guangxi Province.

### Sequence Data

GenBank accession numbers for the sequences reported in this study are KR106771-KR106986 for the partial *pol* gene.
